# Adolescent smoking and tertiary education: opposing pathways linking socio‐economic background to alcohol consumption

**DOI:** 10.1111/add.13365

**Published:** 2016-05-09

**Authors:** Michael J. Green, Alastair H. Leyland, Helen Sweeting, Michaela Benzeval

**Affiliations:** ^1^ MRC/CSO Social and Public Health Sciences UnitUniversity of GlasgowGlasgowUK; ^2^ Institute for Social and Economic ResearchUniversity of EssexColchesterUK

**Keywords:** Alcohol, education, life‐course, pathways, smoking, socio‐economic position

## Abstract

**Background and Aims:**

If socio‐economic disadvantage is associated with more adolescent smoking, but less participation in tertiary education, and smoking and tertiary education are both associated with heavier drinking, these may represent opposing pathways to heavy drinking. This paper examines contextual variation in the magnitude and direction of these associations.

**Design:**

Comparing cohort studies.

**Setting:**

United Kingdom.

**Participants:**

Participants were from the 1958 National Child Development Study (NCDS58; *n* = 15 672), the British birth cohort study (BCS70; *n* = 12 735) and the West of Scotland Twenty‐07 1970s cohort (T07; *n* = 1515).

**Measurements:**

Participants self‐reported daily smoking and weekly drinking in adolescence (age 16 years) and heavy drinking (> 14/21 units in past week) in early adulthood (ages 22–26 years). Parental occupational class (manual versus non‐manual) indicated socio‐economic background. Education beyond age 18 was coded as tertiary. Models were adjusted for parental smoking and drinking, family structure and adolescent psychiatric distress.

**Findings:**

Respondents from a manual class were more likely to smoke and less likely to enter tertiary education (e.g. in NCDS58, probit coefficients were 0.201 and –0.765, respectively; *P* < 0.001 for both) than respondents from a non‐manual class. Adolescent smokers were more likely to drink weekly in adolescence (0.346; *P* < 0.001) and more likely to drink heavily in early adulthood (0.178; *P* < 0.001) than adolescent non‐smokers. Respondents who participated in tertiary education were more likely to drink heavily in early adulthood (0.110 for males, 0.182 for females; *P* < 0.001 for both) than respondents with no tertiary education. With some variation in magnitude, these associations were consistent across all three cohorts.

**Conclusions:**

In Britain, young adults are more likely to drink heavily both if they smoke and participate in tertiary education (college and university) despite socio‐economic background being associated in opposite directions with these risk factors.

## Introduction

Socio‐economic inequalities in excessive alcohol consumption are inconsistent in both adolescence 1, 2, 3 and early adulthood 3, 4, 5, 6, which are key developmental periods for drinking 7, 8, 9. Some studies show no relationship, others show positive and yet others negative associations 1, 4. It has been suggested that these inconsistent findings result from pathways associated with socio‐economic position (SEP) working in opposing directions 4; while some pathways leading to increased drinking are more common among more disadvantaged adolescents, others may be more common among more advantaged adolescents. Opposing pathways could result in no association between SEP and drinking, or associations in either direction. Developing a better understanding of the stratification of pathways leading to (heavy) drinking could lead to more effective and targeted interventions or policies to prevent it. This paper therefore explores two probable opposing pathways between parental socio‐economic position and drinking in adolescence and early adulthood—smoking and tertiary education. Analyses are undertaken in three different cohorts to assess how the findings vary across time and place.

### Smoking pathway

Young people from a disadvantaged SEP are more likely to smoke, and to start smoking earlier 10, 11, 12. Smoking, in turn, is often described as a ‘gateway drug’, associated with onset of alcohol use and alcohol problems 13. Previous analysis of West of Scotland data (also analysed here) found that late adolescent heavy drinkers from disadvantaged backgrounds tended to have smoked prior to drinking heavily, whereas those from more advantaged backgrounds had rarely smoked 14. This suggests that smoking may be a pathway operating more frequently among those from a disadvantaged socio‐economic background, although it is not yet clear whether this pattern extends into early adulthood or whether it would be replicated in other contexts.

### Tertiary education pathway

The second pathway examined here is tertiary education (meaning post‐secondary school education undertaken for example in universities or further/vocational education colleges). Young people from more advantaged backgrounds are more likely to enter tertiary education 15, and students in tertiary education drink more heavily than similar‐aged peers outside tertiary education 16, 17, 18. Thus, tertiary education could be a pathway promoting heavier drinking which operates more frequently among those from a more advantaged socio‐economic background.

### Contextual variation

Contextual heterogeneity may occur either in the associations between SEP and these mediating factors (smoking and tertiary education) or in the associations between those mediators and drinking. Therefore, these pathways are explored with data from three different cohorts: the UK 1958 National Child Development Study (NCDS58), the 1970 British Birth Cohort Study (BCS70) and the 1970s cohort of the West of Scotland Twenty‐07 Study (T07). NCDS58 and BCS70 include people from across Great Britain born 12 years apart, comparing different historical contexts within the same geographical area. T07 respondents were from approximately the same time‐period as BCS70, but from the specific geographic context in and around Glasgow, a large urban city which had been experiencing rapid deindustrialization.

Variation in associations between SEP and mediating factors might be expected in the United Kingdom between the two time‐periods examined, as labour markets shifted from manual to non‐manual occupations 19, 20 and income distributions became more unequal 15, 21. Thus, some indicators of SEP may indicate greater relative disadvantage in more recent cohorts, so stronger associations might be expected between background SEP and outcomes such as smoking or tertiary education. Additionally, stronger associations between background SEP and smoking and tertiary education might be expected in T07: smoking, as a coping mechanism or feature of social life, may have been especially likely for young people from disadvantaged backgrounds here as manual industries declined 19 and jobs became concentrated in the South of the United Kingdom 21; Scotland has also tended to have higher overall rates of participation in tertiary education than elsewhere in the United Kingdom, but with wider inequalities 22.

Heterogeneity might also be expected in associations between these mediating factors and drinking. Alcohol has become more available in the United Kingdom between the two time‐periods examined 23, increasing opportunities for consumption. If smoking or tertiary education increase individual motivation to drink, the association may be stronger in more recent cohorts, where motivations could be acted upon more easily. This strengthening of association might still be expected despite temporal trends in the prevalence of these mediating factors, such as declining smoking rates 23 or increasing participation in tertiary education 15, assuming that a change in the prevalence of the mediator does not change the nature of its effect on drinking motivation.

### Aim and hypotheses

The aim of this paper is to investigate two pathways (smoking and tertiary education) between SEP and drinking in adolescence and early adulthood. We compare three UK cohorts representing different historical and geographical contexts. Specifically, we hypothesize that:

- a disadvantaged socio‐economic background will be associated with a higher likelihood of adolescent smoking which, in turn, will be associated with heavier drinking in adolescence and early adulthood;
- an advantaged socio‐economic background will be associated with a higher likelihood of participation in tertiary education which, in turn, will be associated with heavier drinking in early adulthood;
- socio‐economic background will be associated more strongly with smoking and tertiary education in more recent cohorts, and most strongly in T07; and
- smoking and tertiary education will be associated more strongly with drinking in the two more recent cohorts.

## Methods

### Participants

NCDS58 follows children born within Great Britain in 1958 24. This paper primarily uses data from follow‐up surveys in adolescence (mean age = 16.0 years) and early adulthood (mean age = 23.6 years, in 1981), although data from earlier surveys were also used for weighting and imputation. A total of 15 672 respondents had valid data for analysis, having participated in either the adolescent or early adult follow‐up. This comprised 84.4% of the total sample of 18 558 (17 415 baseline respondents plus 1143 immigrants and others not interviewed at baseline; baseline response rate = 98.8%).

BCS70 is similar to NCDS58, following a cohort born within Great Britain in 1970 (Northern Irish births were also included at baseline but never followed‐up, so excluded here 24). Data were taken primarily from follow‐ups in adolescence (1986; mean age = 16.1 years) and early adulthood (1996; approximate age = 26 years), with data from earlier surveys used for weighting and imputation. Valid data for analysis from either the adolescent or early adult follow‐ups were available for 12 735 respondents (66.7% of the total sample of 18 488; 16 568 at baseline plus 1920 immigrants and others missed at baseline; baseline response rate = 95.8%).

T07 has followed three cohorts of people from in and around Glasgow for 20 years 25. The youngest cohort, analysed here, had a mean age of 15.7 years at baseline in 1987. The baseline sample (*n* = 1515; response rate = 85%) was representative of the population within the sampled area 26, and all cases were included in the analysis. Data were primarily from baseline and a 1994 follow‐up in early adulthood (*n* = 1181; mean age = 21.7 years).

### Measures

Drinking was self‐reported in all cohorts in adolescence and early adulthood. Weekly drinking in adolescence was based on reported alcohol consumption within the last week (NCDS58, BCS70) or drinking frequency (BCS70, T07). In BCS70, data from the question on frequency were preferred over past week consumption (which may have been atypical), but the latter was used if frequency data were missing (*n* = 332). In early adulthood, respondents in all three cohorts reported their past week's drinking, and numbers of alcohol units were derived. Drinking in excess of 14 units for women and 21 units for men 27 was coded as heavy drinking.

All cohort members self‐reported smoking in adolescence. As daily smoking would indicate an established habit, the closest indication of daily smoking available within each cohort was utilized: smoking 10 or more cigarettes weekly in NCDS58; six or more weekly in BCS70; and seven or more weekly in T07. Precise wordings of questions on smoking and drinking are included in Supporting information, Table S1.

Based on detailed histories of economic activity from age 16, respondents were coded as participating in tertiary education if they had reported being in full‐time education after age 18. Background SEP was indicated by parental occupational class, coded according to the British Registrar General's classification 28 as either non‐manual (I, II and III non‐manual) or manual (III manual, IV and V) using the highest status from couple parents. Sensitivity analyses utilized measures of income (contrasting the lowest tertile of equivalized household income with all others) and parental education (contrasting parents who had left school by age 16 with those who remained longer).

Parental smoking, parental drinking, family structure and adolescent psychiatric distress were considered possible confounders. Parental smoking was reported by parents during adolescent surveys (and also reported by adolescents in BCS70). For parental drinking: in NCDS58 interviewers at age 7 indicated whether the family suffered from problems with alcoholism; in BSC70 parent or child reports of a parent drinking ‘three or four times a week’ or more or on ‘most days’ were coded as heavy parental drinking; and for T07 either parent consuming more than 14 units (women) and 21 units (men) was coded as heavy parental drinking. Family structure distinguished between single‐ and two‐parent families. Adolescent psychiatric distress was indicated by the 12‐item General Health Questionnaire in BCS70 and T07, with scores of 3 or more indicating psychiatric distress 29, 30. For NCDS58, psychiatric distress was indicated by scores of 2 or more on the neuroticism component of the Rutter behavioural scale 31.

### Analysis

**Figure 1:**
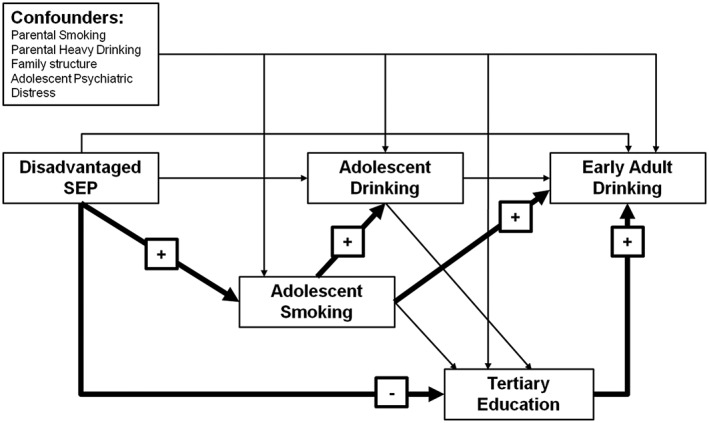
Analysis model and hypothesized direction of effects

Figure 1 depicts the analysis model, tested using structural equation modelling (SEM) in Mplus 7 32. The positive and negative signs indicate the hypothesized directions of association for smoking and tertiary education pathways linking SEP and drinking. The additional associations in the model are not the focus, and hence no specific hypotheses were made about them. Models were estimated using a robust weighted least‐squares estimator (WLSMV) with Probit parameter estimates. Standard errors were calculated with bootstrapping. SEM provides model‐fit statistics for the overall model and facilitates cross‐group comparisons of parameter estimates. A cross‐classified grouping variable based on gender and cohort was used to examine gender and cohort differences. Thresholds for categorical dependent variables were permitted to vary by gender and cohort, and a Wald test was used to examine differences in coefficients (gender differences were tested first, then cohort differences).

Respondents within each cohort had missing data, so multiple‐imputation and inverse probability weighting were employed 33. These adjust for missing values to the extent that they can be predicted by observed variables 34. Weighting adjusted for differences between the analysis and baseline samples of NCDS58 and BCS70, with weights for these cohorts calculated using relevant baseline variables (respondents who were male, had low birth weight, came from single‐parent families or, in BCS70, came from disadvantaged families, were more likely to have dropped out; results not shown). Weighting was unnecessary for T07, as all respondents had some valid data. Multiple imputation (25 imputations) was used to obtain full data on all variables for respondents in the analysis samples within each cohort. Imputation models included additional SEP indicators and variables often associated with smoking and drinking (Supporting information, Table S2 provides details). Weights were included in the imputation models and used to weight the analyses of the imputed data 33.

## Results

### Descriptive statistics and missing data

Table 1 displays descriptive statistics and information on missing data from within each cohort. Adolescent daily smoking was lower in the more recent cohorts. Adolescent weekly drinking was higher in BCS70 than NCDS58 and particularly low in T07. Respondents in NCDS58 were most likely to come from manual households, with little difference between BCS70 and T07 in this regard. Participation in tertiary education was higher in the more recent cohorts and highest in the Scottish cohort. Heavy drinking in early adulthood was highest in T07 and lowest in BCS70 respondents.

**Table 1:** Descriptive statistics and missing data.

		*NCDS58*				*BCS70*				*T07*			
		*15 672*				*12 735*				*1515*			
		*Observed* a		*Weighted and Imputed* b		*Observed* a		*Weighted and Imputed* b		*Observed* a		*Imputed* c	
	*Analysis, n*	*n*	*%*	*n*	*%*	*n*	*%*	*n*	*%*	*n*	*%*	*n*	*%*
Gender	Male	8032	51.3	8102	51.7	6279	49.3	6648	52.2	737	48.6	737	48.6
	Female	7640	48.7	7570	48.3	6456	50.7	6087	47.8	778	51.4	778	51.4
Adolescent measures (age 16)													
Participated in adolescence	No	1307	8.3			2362	18.5			0	0.0		
	Yes	14 365	91.7			10 373	81.5			1515	100.0		
Daily smoking	No	8752	73.1	11 331	72.3	5269	81.1	10 048	78.9	1273	84.5	1280	84.5
	Yes	3217	26.9	4341	27.7	1224	18.9	2687	21.1	234	15.5	235	15.5
	Missing	3703	23.6			6242	49.0			8	0.5		
Weekly drinking	No	6497	54.1	8526	54.4	3068	47.8	6062	47.6	1424	94.3	1429	94.3
	Yes	5509	45.9	7146	45.6	3345	52.2	6673	52.4	86	5.7	86	5.7
	Missing	3666	23.4			6322	49.6			5	0.3		
Parental occupational class	Non‐manual	5538	49.6	7711	49.2	4430	65.3	7475	58.7	891	59.8	904	59.7
	Manual	5633	50.4	7961	50.8	2350	34.7	5260	41.3	598	40.2	611	40.3
	Missing	4501	28.7			5955	46.8			26	1.7		
Parental smoking	No	3232	27.8	4341	27.7	4121	41.8	5145	40.4	398	28.4	408	26.9
	Yes	8377	72.2	11 331	72.3	5734	58.2	7590	59.6	1002	71.6	1107	73.1
	Missing	4063	25.9			2880	22.6			115	7.6		
Parental heavy drinking^d^	No	11 467	98.9	15 500	98.9	6560	68.5	8800	69.1	1162	83.5	1262	83.3
	Yes	124	1.1	172	1.1	3015	31.5	3935	30.9	230	16.5	253	16.7
	Missing	4081	26.0			3160	24.8			123	8.1		
Family structure	Single‐parent	1026	8.8	1395	8.9	561	10.3	1477	11.6	202	13.7	211	13.9
	Two‐parent	10 660	91.2	14 277	91.1	4872	89.7	11 258	88.4	1273	86.3	1304	86.1
	Missing	3986	25.4			7302	57.3			40	2.6		
Adolescent psychiatric distress	No	10 129	82.4	12 851	82.0	3548	71.7	9233	72.5	1193	84.7	1285	84.8
	Yes	2161	17.6	2821	18.0	1402	28.3	3502	27.5	215	15.3	230	15.2
	Missing	3382	21.6			7785	61.1			107	7.1		
Early adulthood measures (aged 22–26)													
Participated in early adulthood	No	3135	20.0			3732	29.3			334	22.0		
	Yes	12 537	80.0			9003	70.7			1181	78.0		
Tertiary education participation	No	9945	79.3	12 538	80.0	6235	70.1	9195	72.2	885	63.7	983	64.9
	Yes	2592	20.7	3134	20.0	2658	29.9	3540	27.8	504	36.3	532	35.1
	Missing	3135	20.0			3842	30.2			126^e^	8.3		
Heavy drinking in early adulthood	No	9366	74.8	11 644	74.3	6935	78.8	9972	78.3	714	61.4	914	60.3
	Yes	3160	25.2	4028	25.7	1861	21.2	2763	21.7	448	38.6	601	39.7
	Missing	3146	20.1			3939	30.9			353	23.3		
Additional information on missing data													
Participated in adolescence and early adulthood	No	4442	28.3			6094	47.9			334	22.0		
	Yes	11 230	71.7			6641	52.1			1181	78.0		
Complete data on all analysis variables	No	9995	63.8			10 252	80.5			581	38.3		
	Yes	5677	36.2			2483	19.5			934	61.7		

a Unweighted data. In order to facilitate comparisons with weighted/imputed data, percentages are based on those with valid responses, except those for missing categories which use the analysis sample as the denominator.

b Percentages are based on weighted average results across 25 imputed data sets.

c Percentages are based on average results across 25 imputed data sets.

d In NCDS58 Parental Heavy Drinking was ascertained by interviewers during interviews at age 7, whereas in BCS70/T07 it was reported at age 16 by parents and/or respondents.

e There are more valid responses than those participating in the early adulthood survey here because supplementary data from an intervening interview at age 18 were also used to indicate participation.

Response rates in adolescence (81.5–100.0%) and early adulthood (70.7–80.0%) were reasonable. However, additional item‐non‐response among participants in each survey (particularly evident in BCS70; attributed to a teachers' strike which coincided with the adolescent in‐school surveys) meant that there were relatively low proportions of respondents with complete data on all analysis variables: 36.2% in NCDS58, 19.5% in BCS70 and 61.7% in T07. Nevertheless, as Table 1 indicates, sample proportions for most characteristics remained similar after weighting and imputation, indicating that missing data rates did not differ substantially in terms of the analysis variables.

**Figure 2:**
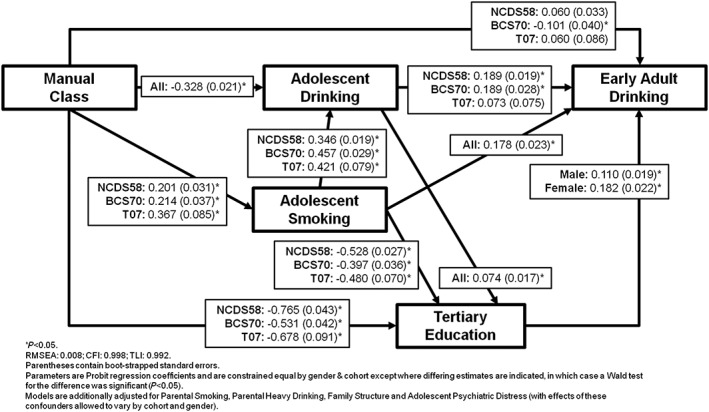
Probit coefficients (and standard errors) from analysis model

Figure 2 shows probit regression coefficients and boot‐strapped standard errors from the confounder‐adjusted analysis model (confidence intervals and *P*‐values are presented in Supporting information, Table S3). Models that did not include confounder adjustment had similar results, although coefficients were a little larger (not shown). Separate estimates are provided where Wald tests for cohort or gender differences were significant (*P* < 0.05). Results from sensitivity analyses with income or education as measures of SEP were largely consistent (see Supporting information, Tables S4 and S5).

### Smoking pathway

Adolescent smoking was more likely for respondents from manual than non‐manual households. The association was stronger in T07 than in NCDS58 and BCS70 (*P* < 0.05, although this cohort difference was not replicated for parental education and income).

Adolescent smoking was associated with more adolescent weekly drinking. This association was stronger in BCS70 and T07 than in NCDS58 (*P* < 0.05). Adolescent smoking was associated independently with heavy drinking in early adulthood, but between‐cohort differences in this association were not significant.

### Tertiary education pathway

Respondents from manual compared to non‐manual households were less likely to participate in tertiary education. The association was strongest in NCDS58 and weakest in BCS70 (*P* < 0.05, although this cohort difference was not replicated for parental education or income). Tertiary education was associated with heavier adult drinking, and this association was stronger for females than for males (*P* < 0.05).

## Discussion

Pathways linking SEP and drinking in adolescence and early adulthood were investigated in three UK cohort studies. In all cohorts, socio‐economic disadvantage was associated with higher chances of smoking in adolescence, and adolescent smoking was associated with heavier drinking in adolescence and early adulthood. However, disadvantaged adolescents were less likely to participate in tertiary education, and tertiary education was also associated with heavier drinking in early adulthood, especially for females. Both pathways leading to heavier drinking were associated with SEP, but operated in opposing directions. Despite some variation in magnitude, these opposing pathways were observed consistently across the three studies, for three different measures of SEP and for males and females, suggesting that they represent consistent phenomena. This analysis does not demonstrate causality, although there are possible causal links (described below).

### Smoking pathway

Associations between adolescent smoking and drinking are consistent with previous research 13, 35 and indicate that smoking may link socio‐economic disadvantage and heavier drinking. This could be, in part, because the physiological effects of nicotine stimulate drinking 36, 37, although reverse causation may contribute to this association if alcohol also disinhibits smoking behaviour. Further, there may be common pathways leading to both tobacco and alcohol use that are associated with socio‐economic disadvantage 14. Smoking and drinking behaviours may both represent coping strategies used by young people as they face the wide range of stressors associated with a disadvantaged SEP 38. Other pathways associated with socio‐economic disadvantage that lead to greater chances of developing both smoking and drinking behaviours may include lack of alternative activities 39, lower quality parental monitoring 38, 40, increased exposure via parents and peers who smoke and drink heavily 3, 41, 42, 43 or a greater likelihood of externalizing behaviour 43, 44, 45. If there are common pathways, it is important to understand their relative importance. Interventions addressing common pathways may be especially effective in tackling both smoking and drinking behaviours among young people from a disadvantaged SEP.

### Tertiary education pathway

Associations between tertiary education and heavier drinking are also observed commonly 16, 17 and may link socio‐economic advantages to heavier drinking in early adulthood. It is presumably not the actual education but experiences associated with it that account for this. Drinking may be a coping response to transitional challenges, may be valued for social goals or overestimation of how much peers drink may inflate perceived behavioural norms 46, 47. However, these factors may also apply to those transitioning into work and other adult roles. Perhaps increasing independence and freedom, combined with low parental monitoring, few adult responsibilities and close involvement with peers in similar situations, contribute to students' higher drinking levels 48, 49, 50. This association was stronger for females than males. Given associations between education and egalitarian gender‐role attitudes 51, tertiary education may have been associated with attenuation of the general population trend (when data were collected) for females in this age group to drink less than males. Heavy drinking rates among UK men and women aged 16–24 converged during the 1990s 52, so the stronger effect of education for females could be historical. If not, it could be increasingly important, as female participation in tertiary education has increased in more recent cohorts 22.

### Residual associations between SEP and drinking

Despite the smoking pathway, results consistently showed more frequent drinking among more advantaged adolescents, prior to entry into tertiary education. This finding suggests that there may be other pathways associated with socio‐economic advantage, besides tertiary education, which lead to heavier drinking. For example, alcohol may be more available in more advantaged homes and families 41, 53.

### Contextual heterogeneity

Hypotheses regarding contextual variation in the strength of smoking and tertiary education pathways were only partially verified: differences in the associations between socio‐economic disadvantage and smoking or tertiary education were only present for parental occupational class, and not replicated in analyses using income or parental education. Additionally, even for parental occupational class the association between background SEP and tertiary education was strongest rather than weakest in NCDS58 (although it was stronger in T07 than BCS70).

There was a stronger association between adolescent smoking and adolescent drinking in the two more recent cohorts, as hypothesized, but if this was due to increases in alcohol availability then it is not clear why a similar difference was not seen for associations with early adult drinking. Perhaps changes in societal availability matter more for those below the legal drinking age, who are generally more constrained in their opportunities for consumption.

### Limitations

Measurement differences may account for some of the differences in findings between the cohorts. The self‐report drinking measures were not ideal. They would not have captured the full complexity of drinking patterns (e.g. episodic binge drinking might not have been well represented), and may have led to under‐reporting. T07, where reports were given in the home (but not with parents present) had lower prevalence of adolescent drinking than NCDS58 and BCS70 where adolescent measures were administered in schools. Age differences in reporting may be important as heavy drinking in early adulthood, especially among students, can be age‐limited, with recovery to moderate levels within a few years 46. BCS70 measurements were at age 26 rather than 22–23 and had the lowest prevalence of heavy drinking. Age was confounded too strongly with cohort to be included in these models, but the consistency of the association between tertiary education and heavy drinking measured at these different ages suggests that it was not just age‐limited drinking. Additionally, the broad heading of tertiary education may mask heterogeneity in patterns between universities and further/vocational education colleges.

### Conclusions

Interventions focused on reducing excess drinking during tertiary education may be effective at reducing the prevalence of heavy drinking in early adulthood but target a population with more advantaged socio‐economic backgrounds, while ignoring those who drink heavily outside tertiary education (who will tend to be more disadvantaged, and have additional health detriments from smoking). Interventions focused on pathways common to smoking and drinking may tend to benefit more disadvantaged young people. Further research might explore a wider range of pathways and contexts in order to understand more clearly the pathways that lead to heavy drinking in different socio‐economic circumstances, and lead towards designing interventions tailored to people's different backgrounds. Pathways for further study might include: availability of alcohol (within the home and society more broadly); family drinking practices; the role of social norms; externalizing behaviour; and challenges associated with transitions into adulthood.

#### Declaration of interests

None.

## Supporting information


**Table S1** Drinking and Smoking questions by cohort.
**Table S2** Additional measures included in imputation models.
**Table S3** Probit Coefficients, standard errors, 95% confidence intervals and *P*‐Values from models with Parental Occupation.
**Table S4** Probit Coefficients, standard errors, 95% confidence intervals and *P*‐Values from models with Parental Education.
**Table S5** Probit Coefficients, standard errors, 95% confidence intervals and *P*‐Values from models with Income.

Supporting info item

Supporting info item

Supporting info item

Supporting info item

Supporting info item
