# Supplementary material for: Adolescent smoking and tertiary education: opposing pathways linking socio‐economic background to alcohol consumption
Source: Addiction. 2016 May 9;111(8):1457–65. doi: 10.1111/add.13365 (PMC4943526; doi:10.1111/add.13365)
Supplement: Supplementary file 1 — Supporting info item [file ADD-111-1457-s001.docx]

Table S1: Drinking and Smoking questions by cohort

|  | **NCDS58** | **BCS70** | **T07** |
| --- | --- | --- | --- |
|  |  |  |  |
| **Daily Smoking in Adolescence** | How many cigarettes do you usually smoke in a week?  None  Less than 1 a week  1-9 a week  10-19 a week  20-29 a week  30-39 a week  40-49 a week  50-59 a week  60 or more a week  *0=less than 10 weekly;*  *1=10 or more weekly* | How many cigarettes do you smoke in a week?  Non-Smoker  One a week  2-5  6-10  11-20  21-40  41-70  71-100  More than 100  *Or, if missing then...*  Since this time last week, how many cigarettes have you smoked?    *0=less than 6 weekly;*  *1=6 or more weekly* | How many cigarettes (including any roll-ups) do you usually smoke in a week?  *0=less than 7 weekly;*  *1=7 or more weekly* |
|  |  |  |  |
| **Weekly Drinking in adolescence** | How long is it since you had an alcoholic drink (beer, wine, spirits, etc.)?  Less than 1 week  2-4 weeks  5-8 weeks  9-12 weeks  Over 12 weeks  Never    *0=more than 1 week;*  *1=less than 1 week* | In the last 12 months, about how often have you had anything alcoholic to drink?  Every day/most days  4-5 times weekly  2-3 times weekly  Once a week  Once a month  Only on special occasions  Never  *0=less than weekly;*  *1=weekly or more*  *Or, if missing then...*  If you have had any alcoholic drink since this time last week, on how many days did you do so?  *0=none; 1=any* | About how often do you drink [alcohol]?  More than once/day  Once/day  4-6 days/week  2-3 days/week  Once/week  Once/fortnight  Once/month  Once/3 months  Once/6 months  Once/year  Less than yearly  Never  *0=less than weekly;*  *1=weekly or more* |
|  |  |  |  |
| **Heavy Drinking in early adulthood^a^** | In the last seven days, that is not counting today but starting from last [name present day of week], how much beer, stout, lager or cider have you had?  In the last seven days how many measures of spirits have you had?  In the last seven days how many glasses of wine have you had? (take 1 bottle=6 glasses)  In the last seven days how many glasses of martini, vermouth or similar drinks have you had?  *Convert answers to units*  *0=less than or equal to 14/21 units;*  *1=greater than 14/21 units* | In the last week I have drunk:  No alcohol at all  (#pints) Shandy  (#pints) Beer/lager  (#pints) low alcohol beers/lagers  (#pints) Cider  (#pints) Low alcohol cider  (#glasses) Wine  (#glasses) Low alcohol wine  (#single measures) Spirits (Gin, Whisky, Vodka, Rum, Brandy)  (#small glasses) Martini/Cinzano/Sherry  (details) Other alcohol drink  *Convert answers to units*  *0=less than or equal to 14/21 units;*  *1=greater than 14/21 units* | Thinking of last week. How much of each of the following did you drink? If it helps, think back over each day to this time last week. Please write the amount in the space against each type of drink.  Beer, lager, cider (pints)  Wine (glasses)  Martini, sherry or port (glasses)  Spirits (whisky, gin, vodka, etc; measures)  Other alcoholic drinks (glasses)  *Convert answers to units*  *0=less than or equal to 14/21 units;*  *1=greater than 14/21 units* |
|  |  |  |  |

Coding used for analysis is summarized in italics.

^a^The threshold was 14 units for women and 21 units for men.
