# Supplementary material for: Adolescent smoking and tertiary education: opposing pathways linking socio‐economic background to alcohol consumption
Source: Addiction. 2016 May 9;111(8):1457–65. doi: 10.1111/add.13365 (PMC4943526; doi:10.1111/add.13365)
Supplement: Supplementary file 2 — Supporting info item [file ADD-111-1457-s002.docx]

##### Supplementary Table 2: Additional measures included in imputation models

|  | **Ages at which additional included measures were taken:** | | |
| --- | --- | --- | --- |
|  | NCDS58 | BCS70 | T07 |
|  |  |  |  |
| *SEP indicators* |  |  |  |
| **Parental occupational class** | 0, 7 and 11 | 0, 5 and 10 |  |
| **Parental education** | 16 | 5, 10 and 16 | 16 |
| **Household income** | 16 | 10 and 16 | 16 |
| **Housing tenure** | 7, 11 and 16 | 5 and 10 | 16 |
| **Parental employment status** |  | 10 and 16 | 16 |
|  |  |  |  |
| *Other variables* |  |  |  |
| **Psychiatric distress** | 7, 11, 16 and 23 | 5,10, 16 and 26 | 16 and 22 |
| **Parental absence** | 16 | 5, 10 and 16 | 16 |
| **Parental smoking** | 16 | 16 | 16 |
| **Parental monitoring** | 16 |  |  |
| **Parental drinking** | 7 | 16 | 16 |
| **Contact with psychiatrists, social work or judicial systems in childhood** |  |  | 16 |
| **Own Smoking** | 23 | 26 | 22 |
|  |  |  |  |
