# Supplementary material for: Adolescent smoking and tertiary education: opposing pathways linking socio‐economic background to alcohol consumption
Source: Addiction. 2016 May 9;111(8):1457–65. doi: 10.1111/add.13365 (PMC4943526; doi:10.1111/add.13365)
Supplement: Supplementary file 4 — Supporting info item [file ADD-111-1457-s004.docx]

**Supplementary Table 4: Probit Coefficients, standard errors, 95% confidence intervals and P-Values from models with Parental Education**

|  | **All (or NCDS58)^a^** | | | | **BCS70** | | | | **T07** | | | |
| --- | --- | --- | --- | --- | --- | --- | --- | --- | --- | --- | --- | --- |
|  | β | s.e. | 95% CIs | P-value | β | s.e. | 95% CIs | P-value | β | s.e. | 95% CIs | P-value |
|  |  |  |  |  |  |  |  |  |  |  |  |  |
| *Adolescent Smoking on...* |  |  |  |  |  |  |  |  |  |  |  |  |
| **Parent(s) Left School by 16** | 0.224 | 0.028 | 0.169, 0.279 | <0.001 |  |  |  |  |  |  |  |  |
|  |  |  |  |  |  |  |  |  |  |  |  |  |
| *Weekly Drinking in Adolescence on...* | | | | | | | | | | | | |
| **Parent(s) Left School by 16** | -0.188 | 0.027 | -0.241, -0.135 | <0.001 |  |  |  |  |  |  |  |  |
| **Adolescent Smoking** | 0.331 | 0.019 | 0.294, 0.368 | <0.001 | 0.447 | 0.027 | 0.394, 0.500 | <0.001 | 0.383 | 0.073 | 0.240, 0.526 | <0.001 |
|  |  |  |  |  |  |  |  |  |  |  |  |  |
| *Tertiary Education on...* |  |  |  |  |  |  |  |  |  |  |  |  |
| **Parent(s) Left School by 16** | -0.920 | 0.032 | -0.983, -0.857 | <0.001 |  |  |  |  |  |  |  |  |
| **Adolescent Smoking** | -0.537 | 0.026 | -0.588, -0.486 | <0.001 | -0.419 | 0.040 | -0.497, -0.341 | <0.001 | -0.539 | 0.075 | -0.686, -0.392 | <0.001 |
| **Weekly Drinking in Adolescence** | 0.097 | 0.017 | 0.064, 0.130 | <0.001 |  |  |  |  |  |  |  |  |
|  |  |  |  |  |  |  |  |  |  |  |  |  |
| *Heavy Drinking in Adulthood on...* |  |  |  |  |  |  |  |  |  |  |  |  |
| **Parent(s) Left School by 16** | -0.051 | 0.044 | -0.137, 0.035 | 0.242 | -0.126 | 0.042 | -0.208, -0.044 | 0.003 | 0.124 | 0.090 | -0.052, 0.300 | 0.167 |
| **Adolescent Smoking** | 0.173 | 0.023 | 0.128, 0.218 | <0.001 |  |  |  |  |  |  |  |  |
| **Weekly Drinking in Adolescence** | 0.187 | 0.018 | 0.152, 0.222 | <0.001 |  |  |  |  |  |  |  |  |
| **Tertiary Education (males)^b^** | 0.096 | 0.019 | 0.059, 0.133 | <0.001 |  |  |  |  |  |  |  |  |
| **Tertiary Education (females)^b^** | 0.168 | 0.022 | 0.125, 0.211 | <0.001 |  |  |  |  |  |  |  |  |
|  |  |  |  |  |  |  |  |  |  |  |  |  |

^a^Coefficients were constrained equal across cohorts, except where a Wald test indicated a significant difference (p<0.05), in which case cohort specific estimates are presented. Models are additionally adjusted for family structure, parental smoking, parental drinking and adolescent psychiatric distress (with those coefficients allowed to vary across cohort and gender groupings).

^b^This was the only coefficient that differed significantly by Gender (p<0.05).

β=Probit coefficient. s.e.=Bootstrapped standard errors.
